# Plant Prebiotics and Their Role in the Amelioration of Diseases

**DOI:** 10.3390/biom11030440

**Published:** 2021-03-16

**Authors:** Amrit Pal Kaur, Sonali Bhardwaj, Daljeet Singh Dhanjal, Eugenie Nepovimova, Natália Cruz-Martins, Kamil Kuča, Chirag Chopra, Reena Singh, Harsh Kumar, Fatih Șen, Vinod Kumar, Rachna Verma, Dinesh Kumar

**Affiliations:** 1 School of Bioengineering and Food Technology, Shoolini University of Biotechnology and Management Sciences, Solan 173229, Himachal Pradesh, India; amikaur007@gmail.com (A.P.K.); microharshs@gmail.com (H.K.); 2 School of Bioengineering and Biosciences, Lovely Professional University, Phagwara, Punjab 144411, India; sonali.bhardwaj1414@gmail.com (S.B.); daljeetdhanjal92@gmail.com (D.S.D.); chirag.18298@lpu.co.in (C.C.); reena.19408@lpu.co.in (R.S.); 3 Department of Chemistry, Faculty of Science, University of Hradec Kralove, 50003 Hradec Kralove, Czech Republic; eugenie.nepovimova@uhk.cz; 4 Faculty of Medicine, University of Porto, 4200-319 Porto, Portugal; 5 Institute for Research and Innovation in Health (i3S), University of Porto, 4200-135 Porto, Portugal; 6 Laboratory of Neuropsychophysiology, Faculty of Psychology and Education Sciences, University of Porto, 4200-135 Porto, Portugal; 7 Sen Research Group, Biochemistry Department, Faculty of Arts and Science, EvliyaÇelebi Campus, Dumlupınar University, Kütahya 43100, Turkey; fatihsen1980@gmail.com; 8 School of Water, Energy and Environment, Cranfield University, Cranfield MK430AL, UK; Vinod.Kumar@cranfield.ac.uk; 9 School of Biological and Environmental Sciences, Shoolini University of Biotechnology and Management Sciences, Solan 173229, Himachal Pradesh, India; rachnaverma@shooliniuniversity.com

**Keywords:** prebiotics, dietary fiber, oligosaccharides, non-digestible carbohydrates, short-chain fatty acids

## Abstract

Prebiotics are either natural or synthetic non-digestible (non-)carbohydrate substances that boost the proliferation of gut microbes. Undigested fructooligosaccharides in the large intestine are utilised by the beneficial microorganisms for the synthesis of short-chain fatty acids for their own growth. Although various food products are now recognized as having prebiotic properties, several others, such as almonds, artichoke, barley, chia seeds, chicory, dandelion greens, flaxseeds, garlic, and oats, are being explored and used as functional foods. Considering the benefits of these prebiotics in mineral absorption, metabolite production, gut microbiota modulation, and in various diseases such as diabetes, allergy, metabolic disorders, and necrotising enterocolitis, increasing attention has been focused on their applications in both food and pharmaceutical industries, although some of these food products are actually used as food supplements. This review aims to highlight the potential and need of these prebiotics in the diet and also discusses data related to the distinct types, sources, modes of action, and health benefits.

## 1. Introduction

Prebiotics are non-digestible carbohydrate (CHO) molecules, including sugar polyols, poly and oligosaccharides, and resistant starches, as well as fiber that have a beneficial role in both the maintenance and progression of gut microflora. Prebiotics are known for their ability to nourish gut microbes present in the gastrointestinal tract (GIT) and substantially improve their metabolic activity, enhancing digestion, nutrient absorption ability, and the immune system, while curbing the growth of pathogenic microbes [1]. These significant improvements show a positive effect on human health [2]. The ability of prebiotics to sustain themselves in acidic environments and remain resistant to distinct digestive enzymes in the small intestine make them an extraordinary tool to boost the growth of beneficial gut microbes that ferment them, leading to the production of short-chain fatty acids (SCFAs), vitamins, and other fragmented molecules [3].

Prebiotics are generally found in different food sources, such as chicory, chia seeds, dandelion greens, flaxseeds, onion, garlic, almonds, artichoke, oats, barley, and many other plants, although they can also be synthesized via enzymatic digestion of complex polysaccharides [4]. Some common prebiotics, such as fructooligosaccharides (FOS), guar gum, galactooligosaccharides (GOS), and inulin, are available on the market, whereas hydrolysed xylan prebiotic products, such as xylooligosaccharides (XOS) are still in the development stage. Because of the health benefits of prebiotics, many pharmaceutical industries have gained interest in using prebiotics and have started manufacturing them at a cost-effective ratio [5]. Nowadays, a synthetic approach involving enzymatic digestion is predominantly used for the synthesis of high-quality prebiotics. However, the utilization of prebiotics differs from microbe to microbe as the diverse gut microbes tend to have distinct nutritional requirements to remain in the GIT. Generally, gut microbes use prebiotics as nutrient sources for their proliferation and metabolic activity [6], so they have been extensively used in food industries as functional food supplements in different preparations [7]. In this sense, this review focuses on providing updated data about the need for prebiotics and covers information related to their various types, sources, modes of action, and health benefits.

## 2. What Are Prebiotics?

Over the past decades, the term “prebiotics” has significantly evolved. The concept of prebiotics was introduced in 1995 as “non-digestible food ingredients, which exhibit beneficial effects on the host by selectively stimulating the growth and proliferation of one or specific bacteria in the colon that substantially improve the health of the host” [8]. During this time, the substances able to improve the number of bacteria, mainly, Bifidobacteria and Lactobacilli, were also considered prebiotics. However, in 2004, the definition was updated to “selectively fermented ingredients that specifically improve the activity and composition of gastrointestinal microflora and provide benefits to host health and well-being”, thus describing the conditions that exhibit beneficial effects on the host. According to this, prebiotics should have the ability to resist host digestion and be fermented by intestinal microflora [9]. Some years later, in 2010, with the development in molecular approaches and cumulative evidence about the density and diversity of bacterial communities, the International Scientific Association for Probiotics and Prebiotics (ISAPP) released a solidarity statement revising the definition of dietary prebiotic as “a selectively fermented ingredient that results in specific changes in the composition and activity of the gastrointestinal microbiota, thus conferring benefit(s) upon host health” [10]. This revised definition involves the non-specific bacterial species, which expands the location of bacterial species from only the colon to the entire GIT length. However, Bindels et al. (2015) proposed the definition of prebiotics as “non-digestible compounds that, through their metabolization by microorganisms in the gut, modulate the composition and/or activity of the gut microbiota, thus conferring a beneficial physiological effect on the host”. This updated definition eliminated the selective fermentation processes and microorganism specificity as prerequisite requirements, but also limited the prebiotic interaction with gut microbiota without involving extra-intestinal habitats, such as the respiratory tract, vagina, and skin [11]. More recently, with the progressive clinical development and latest scientific development, ISAPP in 2017 again updated the prebiotic concept, and defined it as “a substrate, i.e., selectively used by host microorganisms and conferring a health benefit(s) to the host while retaining the microflora-mediated health benefits”. According to this updated definition, prebiotics are not limited to carbohydrates and foods and are no longer restrained to the GIT; instead, they also involve the non-food elements and extra-intestinal tissues, with this updated definition now also being valid for animals [12].

Presently, the well-known prebiotics involve non-digestible carbohydrates, such as FOS, GOS, inulin, and lactulose [13,14,15,16]. Additionally, other non-digestible carbohydrates, such as arabinoxylan, beta-glucans, isomalto-oligosaccharides (IMO), polydextrose, soybean oligosaccharides, XOS, and xylo-polysaccharide (XPS), have also been claimed to exhibit prebiotic potential based on clinical evidence [17], although most scientific literature available concerning prebiotic potential is related to FOS and inulin [18].

## 3. Prebiotics and Dietary Fiber

In general, prebiotics are unique dietary fibers that act as a substrate for beneficial bacteria in the human gut [4,19]. However, not all prebiotics are considered dietary fiber, as not all dietary fibers tend to exhibit prebiotic effects. In 1953, the term “dietary fiber” was coined, but before that few properties such as increasing stool weight, laxative effects, and disease prevention were already associated with fibers [20]. In 2008, the U.S. Food and Drug Administration (US-FDA) and American Dietetic Association (ADA) stated that fibers could be divided into two categories: (i) dietary fibers which involve lignin and non-digestible carbohydrate, are intact in plants, and intrinsic in nature; (ii) functional fibers that are isolated from non-digestible carbohydrates which have beneficial effects in humans [21]. The US-FDA has incorporated the definition of dietary fibers in the formulation and nutrition facts of foods. This concept defines dietary fibers as “synthetic or isolated carbohydrates with three or more monomeric units, that have a beneficial physiological effect on human health” [22]. Currently, dietary fibers play a substantial role in preventing metabolic (cancer, diabetes, and obesity) and cardiovascular (CVD) diseases [20]. However, to date, there is no general definition of dietary fibers, with various definitions being adopted in different parts of the world [23].

In 2009, the Codex Alimentarius Commission defined dietary fiber, and one year later, the Ninth Vahouny Fiber Symposium added some information to the definition mentioned above [24], according to which, undigestible carbohydrates with a degree of polymerisation in the range of 3–9 were included in the category of dietary fibers. They were claimed to exhibit beneficial effects on human health, including the ability to reduce blood glucose and lipid levels, to decrease intestinal transit, and increase stool mass and microbiota fermentability [24]. Some years later, in 2017, Codex Alimentarius Commission made some additions to the definition of dietary fibers, in which the carbohydrate polymers (10 or more monomeric units) were stated as non-hydrolysable through the action of endogenous enzymes and were defined as naturally present in consumable food, thus being placed in the category “edible carbohydrate polymers”. Indeed, carbohydrates polymer are the polymers obtained from raw food materials via chemical, enzymatic or physical means and are known to have beneficial effects on human health confirmed by competent authorities. Synthetic carbohydrate polymers are chemically synthesised polymers which also show beneficial physiological effects on human health affirmed by scientific evidence generated by competent authorities [25].

Conventionally, dietary fibers are classified as soluble and insoluble fibers. The soluble fibers are proclaimed to exert propitious effects on serum lipids, whereas insoluble fibers produce laxative effects with an increase in stool weight. Furthermore, fibers are also categorised according to fermentability and viscosity features, where fermentable fibers are the ones that are readily metabolised via microbiota, while viscous fibers usually form a gel in the GIT. It is noteworthy that there is not a firm classification for different fibers [20]. Some dietary fibers are readily fermentable, such as partially hydrolysed guar gum, Arabic gum, and soluble corn fibers. These are easily fermented in the gut and exert beneficial effects. On the other hand, poorly fermented fibers include cellulose, which provides roughage but not the benefits that prebiotics display [26,27]. Arabic gum consists mainly of arabinose and galactose and some glycoproteins, although there are conflicting reports on their health benefits [28]. Partially hydrolysed guar gum is composed mainly of hydrolysates of guar seeds rich in galactomannan, which is water-soluble (guar gum is discussed in detail in Section 4.1.7).

To gain insight into dissimilarities between prebiotics and dietary fibers, it is essential to state that human endogenous enzymes do not have the ability to break down various glycosidic bonds present in different polysaccharides, such as cellulose, lignin, hemicellulose, pectin, and mucilage. These polysaccharides are not hydrolysed by endogenous enzymes but are partly fermented in the GIT [29]. Few dietary fibers have beneficial effects as they stimulate the activity and growth of gut bacteria potentially associated with well-being and health, therefore acting as prebiotics [30].

The potential of dietary fiber consumption in modifying microbiota has been widely proved. Moreover, it is well-versed that switching between a fiber-rich (>30 g·day^−1^) diet and a meat-based diet often causes a change in bacterial diversity and the production of fermented products, although not enough to exhibit a prebiotic effect. Therefore, it is stated that the consumption of fiber helps to maintain a beneficial effect in humans [31]. Hiel and colleagues reported that the consumption of a diet with inulin-rich vegetables for three weeks substantially improved the levels of *Bifidobacterium* and *Clostridiales* in the gut [32]. Indeed, the physical and chemical structures of dietary fiber impact which microbes will be able to utilize and ferment it. Dietary fibers with complex chemical structures such as those comprising different linkages, sugars, and branching patterns will require the synergistic action of microbial enzymes for complete breakdown. The number of gut microbes capable of fermenting dietary fibers is inversely proportional to the complexity of dietary fibers. For example, many *Bacteroides* species are known to multiply in media containing glucose and xylose while only a few taxa exhibit the potential to utilize xyloglucans for multiplication [33].

## 4. Types of Prebiotics

Generally, non-digestible carbohydrates are considered prebiotic. However, all prebiotics should fulfil the following criteria: (i) they should be resistant to mammalian enzymes and gastric acidity, (ii), they should be susceptible to gut microbes for fermentation, and (iii) they should improve the activity and the viability of beneficial microbes [34]. Different types of prebiotics exhibit distinct health benefits. For example, inulin, GOS, and FOS have long been considered the chief prebiotics. However, various other compounds and dietary fibers have emerged as candidate prebiotics offering multiple health benefits to varying degrees. The following are eight categories of prebiotic dietary fiber that have been evidenced in the literature to provide health benefits to the consumer. The most commonly known prebiotics, their types, sources, structure production, and potential benefits are shown in Table 1.

### 4.1. Synthetic Prebiotics

#### 4.1.1. Fructooligosaccharides (FOS)

FOS are also known by other names, such as oligofructan or oligofructose, as they are low calorie-containing dietary fibers with prebiotic potential [46]. Presently, FOS are considered natural food ingredients due to their various beneficial effects on animal and human health [47]. They are found in the blue agave plant, cereal grains (barley, wheat, oats), vegetables, and fruits (artichoke, asparagus, bananas, garlic, leeks, and onions) [48]. FOS are also proclaimed to be a significant class of bifidogenic oligosaccharides owing to their high production volume [49]. Different pharmaceutical industries have raised their FOS production through zero-waste production, as the waste feedstock is converted into a nutraceutical product due to its prebiotic nature [50]. Oligosaccharide fructans are employed as an alternative sweetener [35]. Different studies have reported that inulin and FOS increase calcium absorption in the gut of both humans and animals [51]. FOS have numerous beneficial properties; they act as a low-intensity sweetener, non-carcinogenic calorie-free dietary fiber, curb the growth of pathogenic bacteria, improve immunity, enhance mineral absorption, decrease cholesterol levels, promote vitamin B complex synthesis, regulate obesity and diabetes, and prevent colon cancer progression [52]. Now, FOS are added as supplements in infant formulas and food products to trigger beneficial gut microbe growth, which further regulates pathogenic microbes [53].

Structurally, FOS are made up of linear chains of fructose joined via β (2-1) bonds, where fructose units could range from 2–60 and terminate as glucose FOS (oligomers of β-d-fructofuranosyl units linked by (21) linkage) [54]. Moreover, in FOS synthesized from sucrose via enzyme-catalyzed transglycosylation, the termination of the individual molecule has a sucrose-containing reduced end [55]. During FOS synthesis from sucrose, the enzyme fructosyltransferase plays a critical role, whereas at a low sucrose concentration, this enzyme exhibits hydrolytic activity [56]. On the other hand, transfructosylating activity is observed when substrate concentration is high [57]. Briefly, this enzyme acts on sucrose and cleaves β-(1–2) linkages and transfers the fructosyl group to the other acceptor molecule, such as FOS and sucrose by releasing glucose as a by-product [58]. FOS synthesized from sucrose encompasses 2-4 fructofuranosyl residues associated with β-(2–1) bonds, having glucose at the terminal end linked by an α-(1–2) linkage [59]. *Aspergillus* spp. and *Aureobasidium pullulans* have been widely exploited for fructosyltransferase enzyme due to their ability to synthesize FOS from sucrose [35]. Indeed, low-cost by-products and agro-wastes are now being increasingly used as a substrate to synthesize sucrose-based FOS [60]. FOS are commercially produced, added as a supplement in various food products, and used as nutraceuticals as they pass through GIT undigested and reach the large intestine where intestinal bacteria ferment them into SCFAs and lactate [31]. In addition, FOS are now available on the market as functional food ingredients because they seem to be an alternative for fat and prebiotic ingredients [6]. Besides this, FOS are also used in ice-cream, jam, and confectionery product production [54].

#### 4.1.2. Galactooligosaccharides (GOS)

Oligolactose, oligogalactose, and oligogalactosyllactose are GOS [61]. The transglycosylation and isomerization of lactulose (cow milk) transform it into GOS [62]. GOS are also prebiotic as they are not enzymatically digested, but are fermented by probiotic *Bifidobacteria*, granting them a bifidogenic potential [20]. GOS are further categorized into two sub-categories, i.e., GOS with excessive galactose at C_3_, C_4_ or C_6_ and GOS synthesized from lactose through enzymatic transglycosylation [63]. In enzymatic transglycosylation, the end product is the amalgam of tri to pentasaccharides with galactose through β (1→3), β (1→4), and β (1→6) linkages [64]. These GOS are also stated as transgalactooligosaccharides (TOS). GOS have been shown to boost the multiplication of *Lactobacilli* and *Bifidobacteria* [65]. In infants, *Bifidobacteria* show high growth upon GOS ingestion [66]. Moreover, *Bacteroidetes*, *Enterobacteria*, and *Firmicutes* also show proliferation in the presence of GOS, although growth is slower than that of *Bifidobacteria* [67]. Lactulose has also been used to form GOS derivatives, as lactulose-derived GOS are also considered prebiotics [68].

GOS were previously synthesized through electrophilic and nucleophilic displacement, but this method is now uneconomical when employed at the industrial scale [69]. Galactosidase and galactosyl-transferase are operative enzymes involved in GOS formation. Galactosyl-transferase has been reported to synthesize GOS in large quantities [70]. However, a catalytic reaction involving galactosyl-transferase for GOS is quite an expensive approach, as it requires nucleotide sugars as a donor [71]. Hence, to reduce costs, oligosaccharides from human milk and globotriose production are commonly used [72]. As galactosidase synthesizes GOS in a low quantity, different approaches have been explored to improve GOS production [73]. The various techniques involved in increasing GOS production include an increase in the number of acceptors and donors in the reaction, lowered water activity, direct shifting of the equilibrium reaction to the endpoint by eliminating the intermediate molecules, and amending the reaction conditions [61]. An in vivo study revealed that GOS supplementation effectively improved lipid metabolism and enriched the microbiota involving *Alloprevotella*, *Bacteroides*, and *Parasutterella* in a mice model [74]. Furthermore, extensive research has been conducted to assess the effect of GOS in gut microbes in older people, and results obtained from the study revealed that consumption of Bimuno^®^ GOS (B-GOS^®^) substantially improved the number of *Bacteroides* as well as *Bifidobacteria* in the gut [66].

#### 4.1.3. Xylooligosaccharides (XOS)

XOS are formed through β-1-4 linkages among xylose molecules [75] and are found in food material, such as bran, fruits, honey, and vegetables [76]. Both *Lactobacilli and Bifidobacteria* possess the ability to hydrolyze the food material digested in the large intestine [77]. In general, XOS are more beneficial than FOS, as they improved the count of *Bifidobacteria* and reduced the count of pathogenic microbes [78]. In vitro studies conducted in a batch experiment have also shown the selective nature of *Bifidobacteria.* Lecerf conducted a parallel, double-blind, and placebo-controlled study of XOS on healthy humans and found that XOS increased the number of *Bifidobacterium* and butyrate production and also improved the activity of α-glucosidase and β-glucuronidase. On the other hand, a reduction in the concentrations of acetate and p-cresol was also observed [79]. The studies highlighted the potential benefits of XOS on human health. These benefits involve the anti-freezing nature, high water activity, non-digestible and non-carcinogenic nature, the positive effect on gut microbiota, and their applicability in pharmaceutical industries [80]. Another 6-week randomized controlled study done with 20 healthy individuals subjected to consuming porridge (150 g) supplemented with 1.2 g XOS daily resulted in an increase in fecal *Bifidobacterium* and *Lactobacilli* counts. In contrast, a reduction in the *Clostridium* count occurred, without any change in the anaerobic bacterial count compared to those who only consumed rice porridge [81].

#### 4.1.4. Fructans

Fructans are natural polymers found in different functional foods, such as artichoke, asparagus, chicory roots, garlic, leek, and onion, and are widely used as prebiotics for improving human health [82]. Structurally, they are formed of a polymer of fructose linked linearly via β2-1 linkages [83]. Fructans improve the gut physiology by enhancing the growth of *Bifidobacteria* and *Lactobacilli* while providing protecting from pathogenic microbes [43]. In addition, the consumption of fructans as prebiotics is able to substantially improve glucose levels and regulate lipid metabolism as well as decrease the level of lipopolysaccharides (LPS) and diacylglycerol (DAG) in the plasma membrane [84].

#### 4.1.5. Isomaltooligosaccharides (IMO)

Isomalto-oligosaccharides (IMO) are obtained following enzymatic treatment of cornstarch with α-amylase, α-glucosidase, and pullulanase and are dissociated into main components, such as isomaltotriose, isomaltose, and panose [85]. In general, IMO comprise glucose monomers formed by α (1-6) glycosidic linkages. The literature shows that IMO positively impacts *Bifidobacteria* and are metabolized by various other microbes [86]. In another study, the synergistic effect of green tea extract (GTE) and IMO was assessed in the production of pro-inflammatory cytokines, visceral adipose tissue, and glycemic and lipid control. The results obtained were positive, revealing an improvement in the levels of glucagon, insulin, and leptin. Moreover, the combination led to a positive effect on microbiota (*Akkermansia muciniphila*, *Bifidobacterium*, *Lactobacilli*, and *Roseburia*) and improved the *Firmicutes*/*Bacteroidetes* as well as the *Prevotella*/*Bacteroidetes* ratio [87].

#### 4.1.6. Soybean Oligosaccharides (SOS)

The oligosaccharides that are found in soybean are termed soybean oligosaccharides (SOS), which involve stachyose and raffinose. These oligosaccharides are not digested by the stomach or intestine enzymes but are hydrolysed by gut microbiota [88]. SOS are efficient in enhancing the proliferation of *Bifidobacteria* present in the large intestine [89]. Hence, they are also stated as bifidogenic and show the same effect as GOS [90]. SOS are also known as α-galactosyl sucrose derivatives, as they are obtained from soybeans [91]. These oligosaccharides are also found in soy germ powder, whose fermentation properties have been assessed with *Lactobacilli* along with inoculums of fecal bacteria [92]. An in vitro study was conducted to evaluate the fermentation and prebiotic effect of soybean Okara on healthy individuals’ fecal microbiota. The results showed an increase in *Bifidobacteria* and *Lactobacilli* growth, inhibiting the growth of harmful bacteria, such as *Bacteroides* and some *Clostridium* species. Furthermore, Okara’s cell wall was challenging to digest in contrast to FOS, signifying the prolonged prebiotic effect compared to other prebiotics [93].

#### 4.1.7. Guar Gum

Guar gum is a biopolymer made up of a linear chain of β-1,4 mannose associated with α-1,6 galactose units obtained from *Cyamopsis tetragonolobus* seeds [92]. It is collected after separating the endospermic portion of the seed from the germ and husk [94]. The endosperm part of the seed is mainly composed of galactomannan and serves as dietary fiber in nutrition [95]. This gum acts as a thickener and stabilizer in several food products, such as salad dressing, sauce, juice, and ice-cream [96]. In addition, guar gam has a high water-binding capacity, making it a valuable food industry product [97]. A study conducted to assess the prebiotic potential of partially hydrolysed guar gum (PHGG) on the diversity and function of gut microbiota in humans found an increase in the number of *Bacteroides*, *Faecalibacterium*, *Fusicatenibacter*, and *Ruminococcus*, along with a decrease in the number of *Blautia*, *Lachnospiracea*, and *Roseburia* [98].

#### 4.1.8. Pectin Oligosaccharides

Pectin is a complex structural molecule composed of galacturonic acid along with abundant polysaccharide [99]. This molecule is further categorized into the following three components, i.e., polygalacturonan (HGA), rhamnogalacturonan I (RG-I), and rhamnogalacturonan II (RG-II) [100]. This pectin oligosaccharide is predominantly found in the cellulosic components and cell walls of vascular plants [101]. All components, such as HGA, RG-I, and RG-II, form pectin by linking to each other through covalent bonding [102]. According to the literature, an enzymatic method is a practical approach to synthesize pectin oligosaccharides [103]. The enzymatic process involves the hydrolysis of apple and citrus pectin in the membrane, which gives rise to oligosaccharides of 3–4 kDa molecular weight [104]. The selectivity of *Bacteroidetes* and *Firmicutes* for growing on pectin (substrate) suggests that pectin and its derivatives will gain significant attention as the basis for prebiotics [105]. Moreover, pectin and its oligosaccharides are useful in promoting the anti-inflammatory potential of commensal microbes present in the colon of humans [45].

#### 4.1.9. Other Polysaccharides

All starch-containing foods and cereal grains naturally contain resistant starch (RS). RS is further classified into four subdivisions based on digestion resistance [106,107], with RS capacity being influenced by the ratio of amylose and amylopectin, granule morphology, and association with other constituent compounds [106]. A study reported the bifidogenic effect of RS as it increased the concentration of *Akkermansia*, *Allobactum*, *Bacteroidetes*, and *Bifidobacteria* species. Another in vitro study conducted in a mice model showed that RS influenced the concentration of SCFAs [108,109,110]. Glucomannans, another neutral polysaccharide, are found in a few plants, such as eastern white pine, orchid, and Konjac/Oncophyllus (a member of the Amorphophallus family). Glucomannan is obtained from konjac and is predominantly used as a food ingredient in Europe [23]. Konjac glucomannan (KGM) flour has various propitious effects, including reducing constipation, improving blood cholesterol, and glycemia. Additionally, konjac glucomannan has also been reported to stimulate the proliferation of beneficial gut microbes. Al-Ghazzewi and colleagues reported that konjac hydrolysate enhanced *Bifidobacterium* and *Lactobacilli* growth compared to inulin present in Ultra-High Temperature (UHT) milk [111]. Numerous studies on KGM have reported a reduction in the count of *Clostridium perfringens* and *Escherichia coli* [111,112,113,114]. Recently, an in vitro study was conducted using Porang glucomannan (PGM) and inulin (positive control), low-density Konjac oligoglucomannan (LKOG), high-density konjac oligo-glucomannan (HKOG), and KGM. The result showed an increase in *Bifidobacterium* and *Lactobacilli* and a decrease in *Bacteroides* count [115].

### 4.2. Sources of Natural Prebiotics

**Figure 1:**
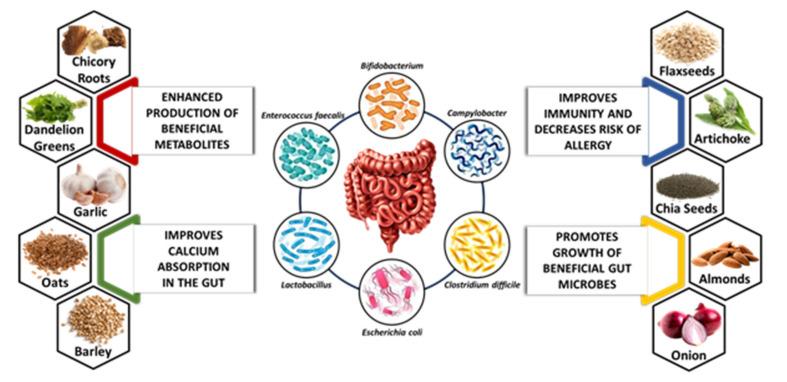
Diagrammatic illustration of sources and functions of prebiotics.

Various non-digestible carbohydrates are naturally found in different plants [116]. The systematic representation of different natural prebiotics and their associated benefits is illustrated in Figure 1.

#### 4.2.1. Dandelion Greens

Dandelion, also known as *Taraxacum officinale*, is a member of the Asteraceae family and a perennial non-poisonous herbaceous weed [117]. It is also proclaimed to be a natural diuretic and is useful in eliminating the body’s excessive retained fluid [118]. Different parts of dandelion plants are being studied for both nutritional and chemical values [119]. Apart from being used as a therapeutic agent, the leaves, roots, and petals of the dandelion plant are used in different food products [120]. For example, the leaves are eaten as a salad in Vietnam and France, either alone or in combination with other plants, such as chives and lettuce [121]. Furthermore, the leaves can be sprinkled with spices, as they are a natural source of calcium, fiber, iron, magnesium, and vitamin A [122]. Dandelion is also rich in oligofructans and other prebiotic fibers [123]. Research has shown the presence of such fibers and has underlined their role in modulating probiotic populations, such as *Bifidobacteria* [124]. Indeed, these fibers are known to enhance the growth of intestinal microbiota and to positively affect lipid metabolism [125]. Dandelion has been used in traditional medicine as well, as a hepatoprotective (liver tonic in Chinese, Indian, and Russian traditional medicine) [126]. Moreover, dandelion roots contain inulin with prebiotic potential, i.e., numerous beneficial effects such as curbing the growth of pathogenic bacteria in the GIT and repressing cancer, obesity, and osteoporosis [127]. However, the inulin content varies with seasons, for instance, 2% secondary compounds were measured in the spring season, whereas 40% was recorded in the autumn season [128].

#### 4.2.2. Chicory Roots

Another *Asteraceae* family member is chicory, also known as *Cichorium intybus*, which has excellent medicinal value [129]. Fresh chicory contains inulin (68%), sucrose (14%), protein (6%), cellulose (5%), ash (4%), and other compounds (3%) in contrast to dried chicory, which contains inulin (98%) and other compounds (2%) [130]. Other than phenolic compounds, chicory leaves also contain minerals (calcium, phosphorus, and potassium) and vitamins (A and C) [131]. Primarily, inulin is the non-digestible prebiotic found in chicory root, which is the polymer of fructose linked through the β (2-1) glycosidic linkage, and it aids in nourishing probiotic bacteria [132]. Although inulin shares a similarity with FOS, their chemical structure is quite distinct as molecular chains of FOS are shorter than those of inulin [133]. Nowadays, inulin is used to replace sugar and fat in different food products [134].

#### 4.2.3. Chia Seeds

Chia is also known as *Salvia hispanica*, a Lamiaceae family member, and an annual herbaceous plant [135]. The seeds are rich in proteins and fats, predominantly rich in various exogenous amino acids and dietary fibers [136]. Considering only the dietary fiber content, chia seeds surpass cereals, dry fruits, and nuts [137]. Defining the features of chia seeds, they contain chiefly α-linolenic acid (polyunsaturated fatty acids), which accounts for 60% fatty acids, whereas other fatty acids, such as oleic, palmitic, and linoleic acids are found in significantly lower amounts [138]. A study reported the effect of crude chia mucilage on the growth of gut microbes and showed that the concentration of chia mucilage did not affect the gut’s physical characteristics (viscosity) but did affect the growth of colonic microorganisms [139]. Another study reported that incorporating chia seeds in the diet can directly enhance gut health and functionality, as well as increase the absorption of zinc and iron [140].

#### 4.2.4. Artichoke

Artichoke, also known as *Cynara scolymus*, is another food with medicinal value [141]. Artichoke is composed of carbohydrates (6.8%) and nitrogen compounds (2.9%), with a high fiber content and low caloric value [142]. Moreover, it also contains other minerals, such as calcium, potassium, and sodium, and in less abundance, iron, manganese, magnesium, copper, and phosphorus [143]. Oligomers are predominantly found in artichoke, which are non-digestible by gastric enzymes but are absorbed in the small intestine after reaching the colon and show a prebiotic effect by promoting the growth of probiotic microbes [42]. It also contains polyphenols and inulin, which are known to exhibit anticancer, antioxidant, and hepatoprotective activities [144]. It has also been proposed to use inulin from artichoke as a prebiotic source with probiotic microbes to develop symbiotic food products [145]. Indeed, these prebiotics markedly increase probiotic viability during production, storage, and in vitro digestion process [146].

#### 4.2.5. Garlic

Garlic, scientifically known as *Allium sativum*, has been used to treat various diseases such as the flu and GI disorders [147]. It is highly rich in FOS, which contribute to the protection of GIT and the prevention of various diseases. Garlic fructan (GF) is one of the significant components of garlic, accounting for nearly 75% of its dry weight, and has been reported to possess prebiotic potential and to influence gut microbiota. A study evaluating the effect of GF on gut microbiota revealed that GF selectively stimulates the *Bifidobacteria* proliferation while represses the less desirable *Clostridia* species, which can support the growth of other pathogens [148].

#### 4.2.6. Almonds

Almonds (*Amygdalus communis* or *Prunus amygdalus*) are a member of the Rosaceae family and belong to *Prunus* species [149]. In almonds seeds, the primary storage component is lipids, which account for the 50% weight of seeds, whereas protein and dietary fibers account for 25% and 12%, respectively [150]. They are considered an excellent source of arginine, monounsaturated fatty acids (MUFA), magnesium, polyunsaturated fatty acids (PUFA), and vitamin E [151]. They also contain a substantial amount of indigestible carbohydrate and unsaturated fats (mono and poly), dietary fiber, vegetable proteins, vitamins, polysterols, polyphenols, and other nutrients that influence the gut microbiome. The skin of almonds is also said to have numerous nutritional benefits as it has a high content of dietary fiber and polyphenols [152]. A study evaluated the prebiotic potential of almond seeds and assessed the impact on the metabolic activity and composition of the gut microflora. Almonds seeds were found to improve the growth of *Eubacterium rectale* and *Bifidobacteria* and produced a high prebiotic index of 4.43 [153]. Another study addressing the effect of almonds on gut microbiota showed an enhancement in *Lactobacillus* spp. and *Bifidobacterium* spp. count and a marked decrease in the proliferation of pathogenic species, such as *Clostridium perfringens*. Indeed, changes in the gut microbiome composition lead to variations in bacterial enzyme activities, including a decrease in nitroreductase, azoreductase, and β-glucuronidase activities and an increase in β-galactosidase activity [154]. An in vivo study analyzing the prebiotic effect of roasted and pre-digested raw almonds revealed the promotion of the growth of *Bifidobacterium breve* (JCM 1192) and *Lactobacillus acidophilus* (La-14) but a decrease in the proliferation of *Enterococcus* spp. Moreover, raw almonds were found to significantly enhance the activity of β-galactosidase and intestinal lipase while lowering the activity of azoreductase and β-glucuronidase [155].

#### 4.2.7. Flaxseeds

Linseed is the other name for flaxseed (*Linum usitatissimum*) [156]. These seeds are considered functional food because they are rich in nutrients and provide health benefits [157]. They comprise various functional ingredients, such as minerals, soluble fibers, high-quality protein, phenolic compounds, and α-linoleic acid [158]. A study showed that the consumption of flaxseed could modify the colon’s microenvironment, significantly enhancing the proliferation of *Prevotella* spp. up to 20 times while repressing the growth of *Akkermansia muciniphila* by 30 times [159]. Another study showed that flaxseed consumption can decrease the growth of *Porphyromonadaceae* and *Proteobacteria* in the gut and may also positively affect the alcoholic liver condition [160].

#### 4.2.8. Onion

Onion, also known as *Allium cepa*, is a member of the Liliaceae family [161]. It not only has nutritional value, but also has medicinal properties [162]. For example, the consumption of onion provides carbohydrates, dietary fibers, vitamins, and minerals [163]. Monosaccharides (glucose, fructose, and sucrose) and FOS are the chief soluble carbohydrates found in the dry matter of onion [164] and show excellent prebiotic effects by improving the health of gut microflora [165].

#### 4.2.9. Oats

The scientific name of oats is *Avena sativa* and is a rich source of polysaccharides (non-starch) [166]. Oats are considered to be healthy cereal grains as they contain a high amount of fiber, minerals, vitamins, and proteins [167]. Moreover, β-glucan is the chief constituent of soluble non-starch polysaccharides found in oats [168]. The ability of β-glucan to form highly viscous solutions is claimed as a health benefit in the human gut [169]. Moreover, oats also have a beneficial role in dyslipidemia, obesity, hypertension, and insulin resistance [170].

#### 4.2.10. Barley

Barley is known by the name *Hordeum vulgare* and is a member of the *Poaceae* family [171]. It is a crop with a low-fat content and high fiber, protein, and vitamin contents [172]. Cereal grains, such as wheat, barley, and oats, have been assessed as potential probiotic cultures in different food products, such as bread, biscuits, beverages, breakfast cereals, and cereal bars [173]. The fermentation of these cereals via probiotic microbes converts them into a digestible form able to boost the proliferation of gut microbes [174]. Barley contains polysaccharides, oligosaccharides, vitamins, and minerals, such as calcium, iron, and zinc [175]. β-glucan is also the main component of barley and exerts immunomodulatory effects by directly or indirectly regulating the gut microbiome [176]. Furthermore, barley has been shown to lower cholesterol levels in the blood, regulate the blood sugar level, and improve immunity [177]. Nowadays, it is being used as raw material for developing functional foods in the food industry [178].

## 5. Mode of Action of Prebiotics

Prebiotics positively influence the proliferation of beneficial gut microflora and their metabolic activities while also improving human health [179]. Generally, prebiotics are resistant to digestion by host enzymes but are readily fermented via gut microbes [180]. They also improve lipid metabolism, which then enhances the absorption of calcium ions that further positively influence bowel and immunological activities [181]. Numerous trials have been conducted on fish to assess the mechanistic action of prebiotics [5]. Specific prebiotics, such as malto-oligosaccharides, GOS, and β-glucans, have been fed to *Channastriata* fingerlings to investigate the effect on their growth, digestibility of nutritional components, regulatory genes of the immune system, and retention properties [182]. The defining mechanism underlying the prebiotic action has not yet been illustrated. However, it is believed that prebiotics can be used by distinct gut microbes as energy and carbon sources depending on their structural and compositional features [96]. Various models have been used to check the effect of prebiotics on different organs of the body [183]. They have been shown to regulate the lipogenic enzymes of the liver, which enhance SCFA production, such as butyric and propionic acids, due to fermentation [31]. These fermented products increase the expression of transcriptional genes, helping in the proliferation of beneficial gut microflora [184]. Prebiotics have advantages over probiotics, as the target bacteria already exist in the host, but it should not be presumed that organisms essential for promoting health are not present in the gut as sometimes prebiotics do not show beneficial effects. Few studies have shown that prebiotics remain ineffective in reducing the number of bacteria such as *Clostridia*, *Bacteroides*, *Enterococci*, and *Enterobacteria* in gut, which have been shown to exhibit detrimental effects on the host health. *Clostridia* species are proclaimed to be toxic as they have the capability to degrade proteins and ferment their amino acids, causing the synthesis of toxic metabolites such as ammonia, amines, H_2_S, thiols, indoles, and phenols, that are involved in colorectal cancer. The sugar composition and polymerization degree of prebiotics along with the available carbohydrates favor *Bifidobacteria* and allow them to proliferate on these substrates [185].

Furthermore, prebiotics, such as FOS modulate mucin production and increase the leukocyte and lymphocyte count in peripheral blood and gut-associated lymphoid tissues (GALTs) [186]. GALTs further aid in the synthesis of immunoglobulin A (IgA), which directly triggers the phagocytic action of intra-inflammatory macrophages [187]. Prebiotics also serve as nutrients for beneficial gut microbes, thereby increasing their abundance at the epithelial level when compared to pathogenic microorganisms by synthesizing certain antimicrobial compounds [188]. Numerous studies have reported the potential effect of prebiotics in modulating cytokine expression. Cani et al. conducted a study to assess the effect of prebiotic carbohydrates on obese mice. The result obtained showed a reduced expression of oxidative stress and inflammatory markers, low profile of plasma LPS, and increased production of pro-inflammatory cytokines (INF-γ, IL-1a, IL-1b, IL-6, and TNF-α) [189]. Similar results have been reported in another study [190].

## 6. Health Benefits of Prebiotics

**Figure 2:**
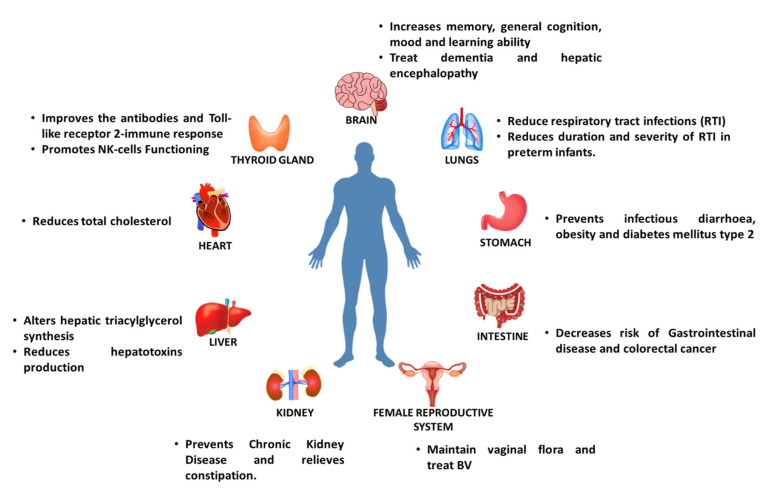
Effects of prebiotics on different organs in humans.

Prebiotics has been shown to exhibit different health benefits in humans (Figure 2).

### 6.1. Effect of Prebiotics on Gut Microbes

A healthy gut microbiome significantly improves the wellbeing and health of individuals [191], which is the primary target for dietary supplements. *Lactobacilli*, a significant gut colonizer, has been reported to decrease gut mucosa inflammation [192], degrade lactose in lactose-intolerant people, relieve constipation, prevent traveler’s diarrhea, and improve irritable bowel syndrome (IBS) [193]. Additionally, *Bifidobacteria* are commonly found in the GIT of healthy humans and are useful in fermenting selective oligosaccharides, making these microbes the usual markers for prebiotic potential [194]. Commensal *Clostridia*, belonging to the phylum Firmicutes, are among the substantial colonizers of the gut, which are known to play a crucial role in modulating immune, physiologic, and metabolic processes [195]. Besides, *C. butyricum* and *A. muciniphila* have been reported to synthesize SCFAs and to exert anti-inflammatory effects. In contrast, some *Clostridium* and *Bacillus* strains have been known to positively influence gut health by constraining the proliferation of pathogenic bacteria [195,196]. Additionally, *A. muciniphila* is believed to exhibit an inverse relationship with diabetes, cardiovascular diseases, low-grade inflammation, and obesity [196]. Prebiotics promote the growth of these beneficial bacteria in the gut and help to boost the immune system activity and treat numerous digestive problems [78]. In addition, they also improve the absorption of calcium and magnesium, control anxiety, enhance bone density, boost the immune system, decrease the triglyceride level in blood, regulate weight and appetite, curb intestinal infection, improve bowel regularity, and reduce inflammation of colon walls [197,198].

### 6.2. Effect of Prebiotics on Metabolite Production

Direct and indirect fermentation of specific compounds generates primary and secondary metabolites, which show health benefits in humans [199]. Microbes present in the gut synthesize SCFAs via fermentation of carbohydrates, amino acids, and other nutrients that are not absorbed in the small intestine [200]. Acetate, butyrate, and propionate are SCFAs that are synthesized after primary anaerobic fermentation of prebiotics by enteric microbes [201]. These SCFAs play a key role as a substrate for cholesterol, glucose, and lipid metabolism. Acetate and propionate act as substrates for peripheral tissues [202]; butyrate plays a vital role as a primary nutrient for colonocytes and serves as a histone deacetylase inhibitor. In addition, as they have ability to inhibit the NF-κB signalling pathway in colonocytes; they contribute to reduce the levels of intestinal inflammation markers and maintain the barrier integrity [203,204]. Other than this, acetate, butyrate, and propionate also boost the G-protein-coupled receptors (GPRs) that modulate the essential metabolic hormones, including GLP-1 and peptide YY (PYY) [205]. Even though SCFA production has numerous positive outcomes, there is a need for extensive research to uncover the real potential [206,207].

### 6.3. Effect of Prebiotics on Mineral Absorption

The primary target of prebiotic consumption is to increase the absorption and bioavailability of calcium to make bones healthy in infants and the elderly [207]. Worldwide, the consumption of prebiotics has reduced the risk of bone fractures and osteoporosis [208]. Calcium is primarily absorbed in the distal intestine, which is stimulated by acidic fermentation of prebiotic dietary fibers as well as chemical changes by numerous microbes [209], although clinical evaluations related to mineral absorption in association with prebiotics have provided mixed results [210]. Few studies related to oligofructose, GOS, inulin, and FOS consumption have shown no significant changes in calcium absorption [211], whereas some studies involving oligosaccharide components with lactulose have shown a significant increase in calcium absorption [212].

### 6.4. Effect of Prebiotics on Allergies

Gut microflora play a crucial role in the development of many disorders. Indeed, a disturbed gut microbiota with reduced microbial diversity can result in many inflammatory and allergic diseases [213]. Various studies suggest that the cause of allergic diseases in the first five years of life is attributed to a reduced colonization of *Lactobacilli* and *Bifidobacteria* in the gut of affected children [214]. Different mechanisms have been listed, all highlighting the immune-modulating effect as well as the importance of dietary oligosaccharides [215]. A hypoallergenic formula containing GOS/FOS supplements has been shown to exhibit protective abilities against allergies, especially against rhinoconjunctivitis and eczema [216]. These reports have shown that infants consuming supplements containing GOS/FOS have a reduced likelihood of developing eczema [217].

### 6.5. Effect of Prebiotics on Diabetes

Diabetes is a complex disease occurring via the interaction between environmental, epigenetic, and genetic factors [218]. Prebiotics play an integral role in the regulation of genes and dramatically impact metabolic functions [219]. Various dietary fibers and carbohydrates generate a link between polymorphisms, which inactivates the insulin-resistant genes [220]. A study conducted on human gut microflora unveiled the interrelation of type 2 diabetes and gut microbiota [221]. Other studies claim the rise inflammatory stress is the cause for the onset of diabetes. Indeed, the daily nutritional diet is believed to be a key factor in the management of diabetes [222]. Studies have suggested that an appropriate diet can significantly decrease the postprandial glucose response [223]. In this way, food items, such as cereals, fruits, spices, and legumes, contain active ingredients such as polyphenols and dietary fibers that aid in decreasing the glycemic index and insulin immune response in patients with diabetes [224]. However, the type of carbohydrates, dosage, and source determine their glucose-reducing effect [225]. For example, inulin-type fructans (ITF) are non-digestible carbohydrate prebiotics with the ability to regulate the growth and composition of gut microbes while confering positive health effects [225]. Arabinoxylan (AX), a prebiotic abundantly found in aleurone fractions and wheat bran, has been reported to undergo fermentation in the colon via beneficial microbes and to positively influence the hyperglycemic levels in diabetic patients [226]. A study conducted on a diabetic mouse model revealed that an increase in the probiotic count in the colon due to supplementation of AX improved insulin resistance [227]. Furthermore, extensive studies are being conducted to understand the impact of AX on gut microbes and to unveil the mechanism of action of AX in lowering diabetic complications [228].

### 6.6. Effect of Prebiotics on Necrotizing Enterocolitis

Necrotizing enterocolitis (NEC) is a gastrointestinal disorder which predominantly affects preterm infants [229], characterized by inflammation, local infection, and necrosis of the bowel in affected patients, leading to high morbimortality rates [230]. Prebiotics, such as GOS and FOS, can trigger the proliferation of beneficial gut microbiota (e.g., *Bifidobacterium*), thus impairing the growth of gut pathogens in premature neonates, ultimately preventing NEC [231]. Additionally, SCFAs have been found to enhance the feeding tolerance in infants by improving the bowel motility and emptying of gastric elements [232].

### 6.7. Effect of Prebiotics on Metabolic Disorders

Irritable bowel syndrome (IBS) is a disorder that adversely affects the intestinal gut microbiota, being directly linked to abnormalities of the mucosa, nervous system, neurotransmitters, immune system, and hormones [233]. Eating habits directly influence the appearance of various symptoms, such as bloating, flatulence, and abdominal pain, which can be curbed by incorporating prebiotics in the regular diet [234]. Several studies claim that the resultant modulation of gut microflora via the addition of prebiotics in the diet reduces such adverse symptoms [235]. A study conducted on wheat bran and guar gum reported that guar gum is more effective than wheat bran in symptom management, such as irregular bowel movements, inflammation, abdominal pain, and epithelial injuries [236]. In addition, a study conducted to analyze the variation in the gut microbiome in IBD patients revealed a reduced count of *Faecalibacterium prausnitzii* observed in the fecal samples of IBD patients. Furthermore, in Crohn’s disease (CD), a type of IBD, it was evaluated that the incidence of a misbalance in gut microbiota was high when *F. prausnitzii* was observed [237]. Prebiotics have been used to control the adverse effects in CD patients [238]. A study reported a decline in the number of *Bacteroides* in the fecal matter of CD patients when they received inulin at 24 g/day [239].

### 6.8. Effect on Hepatic Encephalopathy

Lactulose is considered among the front-line therapeutic agents with effective results in treating hepatic encephalopathy (neuropsychiatric condition), which often results in liver dysfunction. Due to dysfunctional liver, our metabolic system is unable to clear ammonia from the blood stream and it starts accumulating in the liver. The accumulated ammonia, when it reaches a toxic level, has a detrimental effect on the central nervous system. The ammonia is generated by microbiota of the intestine as a protein metabolism end-product. Here, lactulose plays the imperative role by limiting the ammonia synthesis via microbiota and by absorbing the ammonia from the intestinal lumen. Inhibition of deaminating and urease positive bacteria causes the protonation of ammonia to ammonium ions within the intestinal lumen through the acidification of colonic lumen resulting from SCFA [240].

### 6.9. Effect on Female Reproductive Health

*Lactobacilli* species are predominant microbes that are found in the vaginal microenvironment since birth until puberty [241]. After puberty, the diversity of microbes is altered because of hygiene, hormonal changes, menstruation, infections, and intercourse [242]. Owing to this, there is variation in the vaginal environment, which is the reason why lactobacilli bacterial species are not predominant in most women [241]. Due to the non-predominance of *lactobacilli* bacteria in the vaginal environment, there is increased susceptibility to urogenital infections such as bacterial vaginosis and urinary tract infections [243]. Bacterial vaginosis (BV) has been stated to increase the chances of pre-mature delivery and decrease the ability to conceive [244]. The utilization of probiotics has been found effective in reducing the BV risk and preventing pre-mature labor and is supported by a series of both animal and in-vitro studies [245].

## 7. Conclusions

The acceptance of prebiotics as a dietary food ingredient has been found effective in nourishing the gut microbiota. The chemical structure of prebiotics is short-chain oligosaccharides that are fermented by the gut microbiota and enhance their growth. Incorporation of these prebiotics in the diet improves human health and prevents the onset of various diseases. Additionally, beneficial bacteria proliferate and inhibit pernicious bacterial growth and maintain the intestinal balance. They are obtained from various plant sources, but due to their high global demand, the production of such compounds at industrial scales is required. Nowadays, enzymes and microbes are used to increase the availability and variety of prebiotics, such as indigestible carbohydrates, instead of using these compounds in the food industries. Moreover, agro-industrial residues can also be used as alternative substrates for prebiotic production, such as XOS. Synthesized biotics are used to lower the cost of prebiotics on the market and also to improve their quality. In addition, these indigestible carbohydrates can be used as ingredients for preparing different food products, so that the final product has better sensorial and technological features. Nonetheless, to decipher the exact mechanisms behind the beneficial impact of prebiotics on human health is challenging because this depends on the gut microbiota involved in indigestible carbohydrate fermentation that ultimately provides health-promoting functions. Another advantage of prebiotics is their texture-forming ability, which allows them to be used as replacements for fat or sugar because of their exceptional organoleptic quality. Thus, prebiotics can be used to produce various added-value food products due to their bifidogenic properties, ultimately enabling food industries to create new functional foods with unique ingredients, which will positively be accepted by consumers because of the associated health benefits. Also noteworthy is the symbiotic formulation, a different area in this field that remains unexplored. In this approach, a different combination of prebiotics and probiotics can be developed to vary the degree of the therapeutic effect. Thus, studies of these formulations at the nutrigenomics level should be performed to provide deep insights into the individual response to different nutrients.

## Figures and Tables

**Table 1:** Types, structure, production, and potential benefits of prebiotics.

Types of Prebiotics	Chemical Structure	Production Methods	Potential Benefits	Reference
Fructooligosaccharides (FOS)	Glucose and Fructose units linked by β (2→1) glycosidic linkages	Polymerization of fructose monomers	Improve mineral absorption, decrease triglycerides, improve immunity, inhibit pathogenic microorganisms, prevent cancer, and control diabetes	[35,36,37]
Galactooligosaccharides (GOS)	Galactose and Glucose bound by β (1→3) and β (1→4) linkages	Transgalactosylation of lactose using β-galactosidase	Increase bifidogenic activity	[4,38]
Xylooligosaccharides (XOS)	xylose units linked through β (1→4) bonds	Enzymatic hydrolysis of plant xylans	Non-carcinogenic nature, exhibit a positive effect on the intestinal flora, non-digestibility	[39,40]
Soybean oligosaccharides (SOS)	galactose α-(1-6) linked to glucose (Raffinose)galactose α-(1-6) linked to terminal galactose (Stachyose)	NS	Increase the level of IgG, modulate body weight and the immune system	[41]
Isomalto-oligosaccharides (IMO)	Glucose bonds by α (1→4) type	Transglucosylation of liquefied starch	Improve gastrointestinal flora	[42]
Fructans	fructose with β (2→1) linkage	Enzymatic hydrolysis using Fructozyme L	Modulate gut physiology to provide protection from pathogens, improve the level of glucose	[43]
Guar gum	β-D-mannopyranosyl (1-4) linked with α-D-galactopyranosyl (1-6) residues	Enzymatic hydrolysis using cellulase	Improve cholesterol, glycemia	[44]
Pectinoligosaccharides (POS)	(1-4)-α-D-GalA (galacturonic acid) -(1,2)-α-L-Rha	Enzymatic hydrolysis by pectinase	Anti-inflammatory effect	[45]

NS, not specified.
